# *Ligusticum chuanxiong*: a chemical, pharmacological and clinical review

**DOI:** 10.3389/fphar.2025.1523176

**Published:** 2025-04-01

**Authors:** Yin Wang, Liuyun Wu, Hulin Wang, Mingyu Jiang, Yu Chen, Xingyue Zheng, Lian Li, Qinan Yin, Lizhu Han, Lan Bai, Yuan Bian

**Affiliations:** ^1^ Department of Pharmacy, Sichuan Academy of Medical Sciences and Sichuan Provincial People’s Hospital, School of Medicine, University of Electronic Science and Technology of China, Chengdu, China; ^2^ Personalized Drug Therapy Key Laboratory of Sichuan Province, Sichuan Academy of Medical Sciences and Sichuan Provincial People’s Hospital, School of Medicine, University of Electronic Science and Technology of China, Chengdu, China; ^3^ School of Pharmacy, North Sichuan Medical Collage, Nanchong, China; ^4^ Power China Chengdu Engineering Corporation Limited, Chengdu, China; ^5^ Department of Pharmacy, Chengdu Women’s and Children’s Central Hospital, School of Medicine, University of Electronic Science and Technology of China, Chengdu, China; ^6^ Department of Pharmacy, The Fourth People’s Hospital of Chengdu, Chengdu, China

**Keywords:** ethnopharmacology, *Ligusticum chuanxiong*, traditional Chinese medicine, metabolite, pharmacological effects, cardio-cerebral vascular diseases

## Abstract

**Ethnopharmacological Relevance:**

The dried rhizome of *Ligusticum chuanxiong* S.H.Qiu, Y.Q.Zeng, K.Y.Pan, Y.C.Tang and J.M.Xu (*Apiaceae*; including the horticultural variety *Ligusticum chuanxiong Hort*.) [synonym: *Conioselinum anthriscoides* (H.Boissieu) Pimenov and Kljuykov (The taxonomic classification has been adopted by the World Checklist of Vascular Plants)] is a traditional Chinese botanical drug renowned for its anti-inflammatory and antioxidant properties. It has been widely used to treatment various diseases, particularly cardio-cerebral vascular diseases (CCVDs).

**Aim of the review:**

This review aims to summarize recent advances in *Ligusticum chuanxiong* (CX) research, including its chemical composition and pharmacological effects, and modern clinical applications.

**Materials and methods:**

A systematic literature search was conducted using keywords such as “Chuanxiong,” “traditional Chinese medicine,” “chemical components,” “metabolites,” “CCVDs,” and “pharmacological effects” to identify relevant literature published between 2014 and 2025. Databases including PubMed, Web of Science, Google Scholar, and CNKI were utilized. Chemical structures in SMILES format were retrieved from the PubChem, and two-dimensional chemical structures were generated using ChemDraw Ultra 8.0. Classical prescriptions of chuanxiong were obtained from authoritative traditional Chinese medicine databases.

**Results:**

Over 100 metabolites have been isolated and identified from CX, classified into nine major classes. Key bioactive compounds include senkyunolide A, ligustilide, tetramethylpyrazine (TMP), and ligusticum CX polysaccharides (LCP). CX demonstrates significant pharmacological effects in treating CCVDs, such as atherosclerosis (AS), myocardial and cerebral ischemia-reperfusion injury, and hypertension. Its therapeutic mechanisms include antiplatelet activity, endothelial cell protection, anti-inflammatory, antioxidant, and anti-apoptotic properties. CX can be administered alone or in combination with other traditional Chinese medicines (TCMs) or chemical drugs, showing efficacy in cardiovascular, nervous system, digestive system disorders, as well as analgesia and anticancer activities.

**Conclusion:**

CX holds substantial clinical value for treating multi-system diseases, with extensive evidence supporting its use in CCVDs. Further research and clinical exploration of CX are warranted to fully harness its therapeutic potential.

## 1 Introduction

**FIGURE 1:**
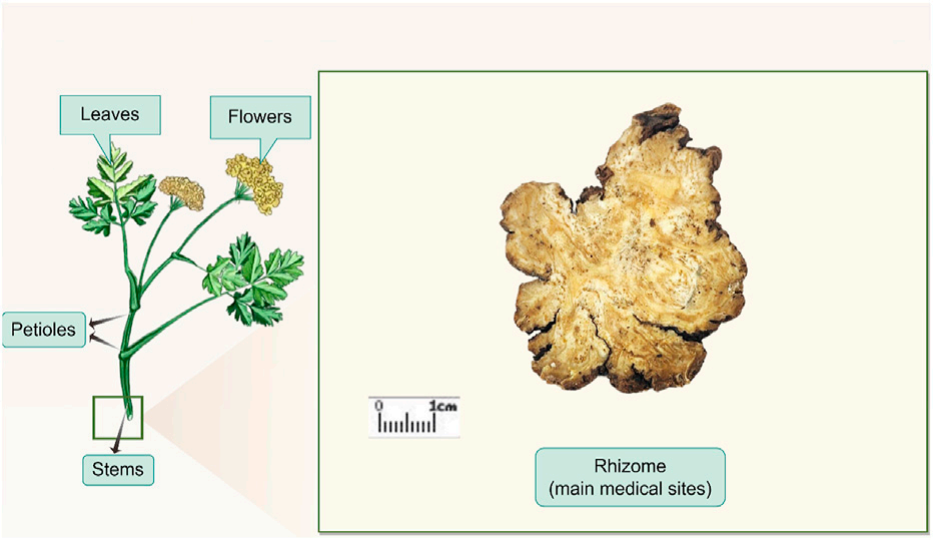
Schematic diagram of *Ligusticum chuanxiong* plant and its medicinal rhizome.

The dried rhizome of *Ligusticum chuanxiong* S.H.Qiu, Y.Q.Zeng, K.Y.Pan, Y.C.Tang and J.M.Xu (Apiaceae; including the horticultural variety *Ligusticum chuanxiong Hort*.) [synonym: *Conioselinum anthriscoides* (H.Boissieu) Pimenov and Kljuykov (The taxonomic classification of this species has been adopted by the World Checklist of Vascular Plants (WCVP))] (CX, Figure 1) is a Chinese herbal medicine first documented in the *Shennong Materia Medica* and further referenced in ancient books such as the *Compendium of Materia Medica* and the *Synopsis of Prescriptions of the Golden Chamber* (Qiu et al., 1979). Table 1 summarizes the definitions and descriptions of *Ligusticum chuanxiong* as outlined in various national or regional pharmacopoeias.

**TABLE 1:** Definition and description of *Ligusticum chuanxiong* in different pharmacopoeias.

Country/Region	Drug name	Species name	Common name	Comment
China	Chuanxiong Rhizome	*Ligusticum chuanxiong* Hort	川芎	
America	Sichuan Lovage Rhizome	*Ligusticum striatum* DC. (syn. *Ligusticum chuanxiong* S.H.Qiu, Y.Q.Zeng, K.Y.Pan, Y.C.Tang and J.M.Xu)	Chuanxiong	
Europe	Ligusticum Root and Rhizome	*Ligusticum sinense* Oliv. or *Ligusticum jeholense* (Nakai and Kitag.) Nakai and Kitag	Ligusticum Root and Rhizome	The species name refers to ligusticum (藁本) in the Chinese Pharmacopoeia
Japan^a^	Cnidium Rhizome	*Cnidium officinale* Makino	センキュウ	
Korea^a^	Cnidium Rhizome	*Cnidium officinale* Makino or *Ligusticum chuanxiong* Hort	천궁(川芎)	
Taiwan	Chuanxiong Rhizome	*Ligusticum chuanxiong* Hort	川芎	
HongKong	Rhizoma Chuanxiong	*Ligusticum chuanxiong* Hort	川芎; chuanxiong	

^a^ Due to significant controversies surrounding *Cnidium officinale* Makino in the Japanese and Korean Pharmacopeias, we have not included pharmacological literature related to the source plants listed in these pharmacopeias.

CX is typically harvested during the summer when stem nodes are visibly swollen and exhibit a slight purple hue. After removal of soil and sand, the rhizomes are sun-dried, roasted, and then cleaned of root hairs. The identification of CX involves morphological, microscopic, and physicochemical analyses. Morphologically, CX appears as irregular, nodular masses with a yellow-brown, rough surface marked by parallel raised nodes and small, tumor-like root scars. The rhizome is firm, emitting a fragrant aroma, and possesses a bitter, spicy taste with a mild numbing sensation. Microscopically, CX is characterized by its cellular structure: the cork layer consists of more than ten rows of cells, with a narrow cortex containing scattered vascular bundles and a distinct cambium. The phloem is broad, and the cambium forms wavy rings or irregular polygons. The xylem vessels are polygonal or round, and the pith is notably large. Parenchyma cells contain oil cells, starch grains, and calcium oxalate crystals. The powdered form of CX is light yellow-brown or gray-brown, with oval or elliptical starch grains and round or cluster-like calcium oxalate crystals. Physicochemical identification involves specific chemical reactions, for instance, when CX powder is treated with petroleum ether and methanol, a red-purple color reaction is observed (Commission, 2020).

**FIGURE 2:**
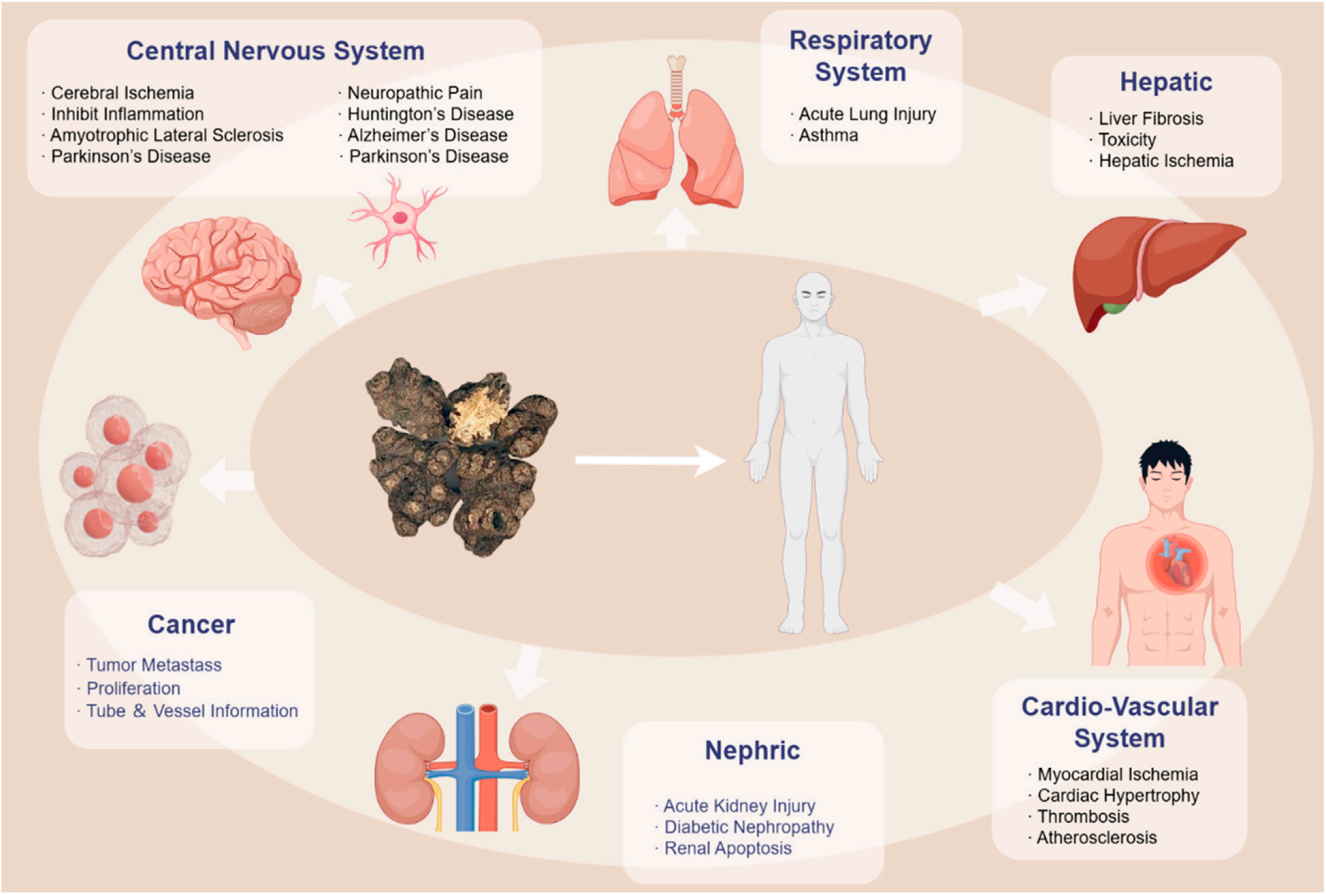
Pharmacological Effects of *Ligusticum chuanxiong*. In TCM theory, CX is a kind of Qi medicine that can reach and work in multiple tissues and organs with the blood. This figure is based on the main affecting parts of the body.

**FIGURE 3:**
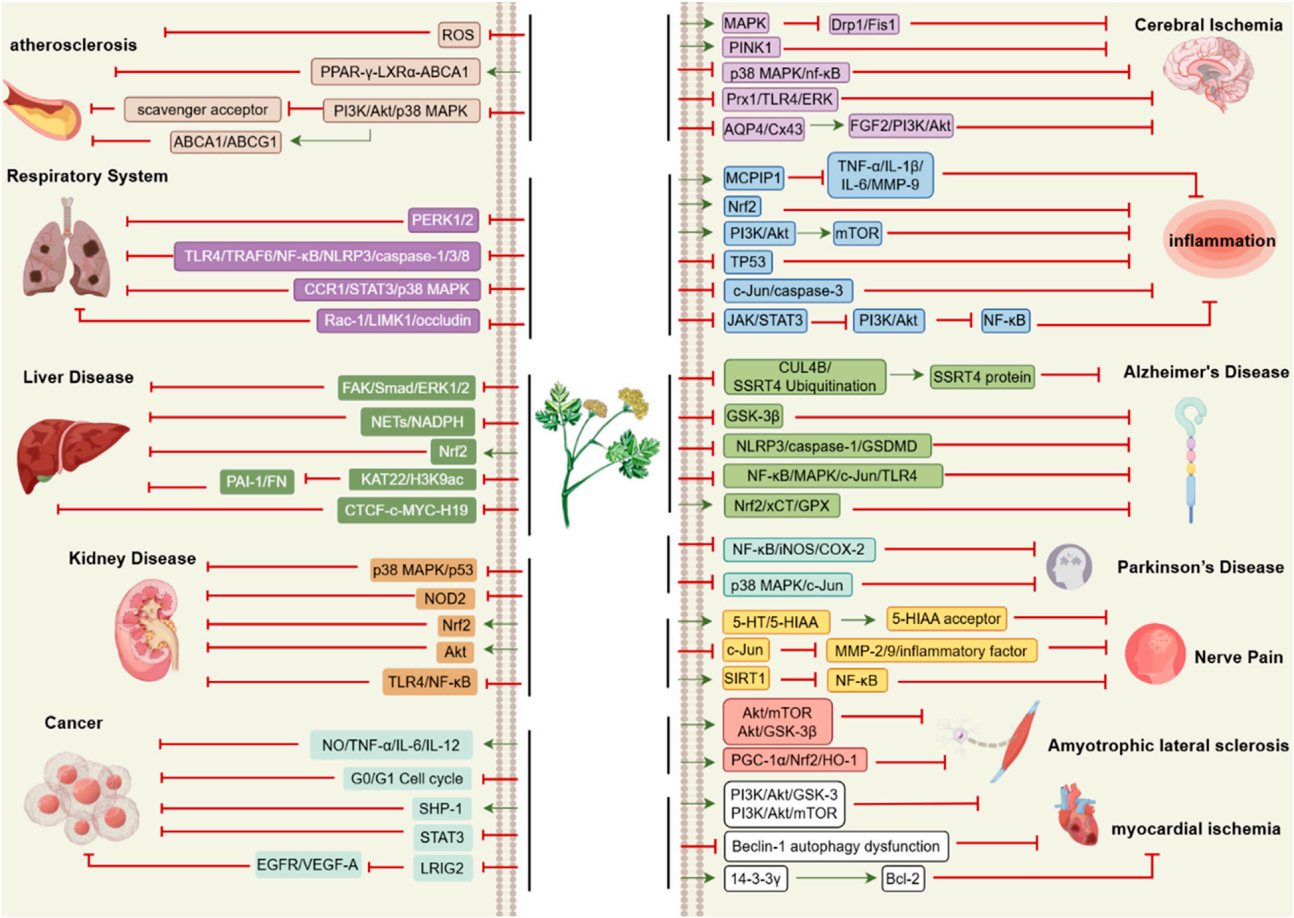
Pharmacological Targets of *Ligusticum chuanxiong*. The specific pharmacological effects of CX are realized through the above pathways. The arrows represent the promoting effects while the dash lines represent the inhibitory effects.

According to TCM theory, CX is characterized by a spicy and warm nature and is associated with the liver, gallbladder, and pericardium meridians. Its therapeutic effects extend upwards to the head and downwards to the *xuehai* point, located in the depression posterior to the ankle joint. CX facilitates blood circulation (活血通络huo xue tong luo), dispels wind-cold (祛风散寒qu feng san han), and exhibits properties such as invigorating blood (活血huo xue), promoting qi flow (行气xing qi), expelling wind (祛风qu feng), and alleviating pain (止痛zhi tong). CX can be used alone or in combination with other Chinese botanical drugs to treat various conditions, including stroke, angina pectoris, bruises, swelling, and menstrual irregularities (Figures 2, 3). Table 2 provides a list of some classic botanical drugs formulas incorporating CX (Chen et al., 2018; Commission, 2020; Shao et al., 2021). Beyond its medicinal applications, CX also holds culinary value, particularly its leaves. The leaves of CX possess a flavor profile reminiscent of celery and yeast, making them suitable for use in soups, stews, or salads. Additionally, the aromatic aerial parts of the plant are often utilized as fragrant botanical drugs (Li Q. et al., 2024; Zhou et al., 2024).

**TABLE 2:** Application of *Ligusticum chuanxiong* in Classical Prescriptions of traditional Chinese medicin**e**.

Prescription name	Main ingredients^a^	Main activities	Clinical application
Chuangxiong Cha Tiao Powder 川芎茶调散	Chuanxiong Rhizome, Notopterygium Root and Rhizome, Dahurian Angelica Root, et al.	Remove wind and relieve pain 疏风止痛 (shu feng zhi tong)	headache due to external wind-evil
Fangdfeng Tong Shen Powder 防风通圣散	Divaricate Saposhnikovia Root, Chuanxiong Rhizome, Chinese Angelica, et al.	Dispersing wind and relieving exterior symptoms, detoxifying heat and relaxing the bowels 散风解表\清热解毒\润肠通便 (san feng jie biao\qing re jie du\run chang tong bian)	Treating all cases of wind-heat
Dachuanxiong Pill 大川芎丸	Chuanxiong Rhizome, Tall Gastrodia Tuber	Dispelling wind and clearing collaterals, promoting circulation of Qi and relieving pain 祛风通络\行气止痛。 (qu feng tong luo\xing qi zhi tong)	head wind and vertigo
Channel-Warming Decoction 温经汤	Medicinal Evodia Fruit, Twig of Chinese Cinnamon Tree, Chuanxiong Rhizome, et al.	Warming menstruation and dispersing cold, dispelling blood stasis and nourishing blood 温经散寒\祛瘀养血 (wen jing san han\qu yu yang xue)	Evidence of deficiency and coldness of the Chong Ren and stagnation of blood stasis
Taohong Siwu Decoction 桃红四物汤	Fresh Rehmannia Root, Tail part of Chinese Angelica Root, Chuanxiong Rhizome, et al.	Promoting blood circulation and removing blood stasis, regulating menstruation and relieving pain 活血化瘀\调经止痛 (huo xue hu ayu\tiao jing zhi tong)	Blood deficiency with Blood stasis
Siwu Decoction 四物汤	Prepared Rehmannia Root, Chinese Angelica, White Peony Root, Chuanxiong Rhizome	Nourishes and regulates blood 补血调血 (bu xue tiao xue)	deficiency and stagnation of camp blood
Constraint-Resolving Pill 越鞠丸	Rhizome of Nutgrass Galingale, Cape Jasmine Fruit, Chuanxiong Rhizome, et al.	relieve melancholy and promote qi circulation 解郁行气 (jie yu xing qi)	six depressive symptoms
Pubescent Angelica and Mistletoe Decoction 独活寄生汤	Pubescent Angelica Root, Asarum Root and Rhizome, Chuanxiong Rhizome, et al.	Dispelling wind-dampness, relieving paralyzing pain, benefiting the liver and kidney, tonifying qi and blood 祛风除湿\通痹止痛\益肝肾\补气血 (qu feng chu shi\tong bi zhi tong\yi gan shen\bu qi xue)	Prolonged paralysis, deficiency of the liver and kidneys, and insufficiency of qi and blood
Toxin-Resolving Powder 败毒散	Notopterygium Root and Rhizome, Pubescent Angelica Root, Chuanxiong Rhizome, et al.	Dispersing cold, dispelling dampness, benefiting Qi and relieving symptoms 散寒祛湿\益气解表 (san han qu shi\yi qi jie biao)	Qi deficiency externally affected by wind-cold-dampness superficial evidence
Blood Stasis-Expelling Decoction 血府逐瘀汤	Peach Seed, Dried Safflower Flower, Chuanxiong Rhizome, et al.	Promoting blood circulation and removing blood stasis, promoting circulation of Qi and relieving pain 活血化瘀\行气止痛 (huo xue hu ayu\xing qi zhi tong)	Evidence of blood stasis in the chest
Engendering and Transforming Decoction 生化汤	Whole Chinese Angelica, Chuanxiong Rhizome, Peach Seed, et al.	Nourishing Blood, dispelling blood stasis, warming menstruation and relieving pain 养血祛瘀\温经止痛 (yang xue qu yu\wen jing zhi tong)	Blood deficiency and cold condensation, blood stasis and obstruction evidence

^a^ Definitions of prescription ingredients are from the Chinese Pharmacopoeia 2020 edition.

China faces a high incidence of CCVDs, which rank among the leading causes of mortality in the population (Liberale et al., 2021; Xu et al., 2022). In recent years, the onset of CCVDs such as hypertension, stroke, and coronary heart disease has shifted significantly to younger age groups, with a notable rise in incidence and prevalence among young and middle-aged individuals. Pharmacotherapy plays a pivotal role in managing CCVDs, with ongoing research and development of new drugs and therapeutic approaches. Conventional Western medications, including nitrates, statins, β-blockers, and antiplatelet agents, have effectively treated these conditions (Sikora et al., 2022). However, patients with CCVDs often face significant residual risks, and Western drugs are associated with varying degrees of adverse effects. Consequently, healthcare professionals are increasingly integrating TCM formulations with conventional Western medications to improve therapeutic outcomes (Li H.-q. et al., 2015; Liu X.-t. et al., 2016).

Chinese herbal medicines that promote blood circulation and resolve blood stasis offer unique advantages in enhancing platelet function and hemodynamics, making them valuable in treating CCVDs (Guo et al., 2020; Li et al., 2022a). As a prominent Chinese herbal medicine, CX has been used in China for centuries. Through the integration of TCM theory and modern pharmacological research, the diverse pharmacological effects of CX have been elucidated, particularly its anti-inflammatory and antioxidant properties. Recent studies have made significant progress in understanding its chemical metabolites, pharmacological effects, pharmacokinetics, and safety profiles. Over 100 chemical metabolites have been isolated from CX, including phthalides, terpenes and their enol derivatives, alkaloids, polysaccharides, and organic acids and their ester derivatives. These metabolites exhibit multiple pharmacological activities, such as vasodilation, blood circulation enhancement, antiplatelet aggregation, antioxidant, and anti-inflammatory effects. Notably, some metabolites, such as ligustilide, have been clinically applied in the treatment of CCVDs (Han, 2017; Chen et al., 2018).

This study conducted a systematic literature search using keywords such as Chuanxiong, traditional Chinese medicine, chemical components, metabolites, CCVDs, and pharmacological effects. Relevant literature published between 2014 and 2025 was retrieved from databases including PubMed, Web of Science, Google Scholar, and CNKI. The inclusion criteria comprised English articles with an impact factor greater than 3.0 and Chinese articles from “*China’s Core Science and Technology Journals*” or “*Peking University Library Chinese Core Journals.*” A total of 8,534 publications were considered to retrospectively review the progress of modern research on CX, summarize its chemical composition and pharmacological effects, and provide a foundation for its contemporary clinical applications.

## 2 Metabolites of *Ligusticum chuanxiong*

**FIGURE 4:**
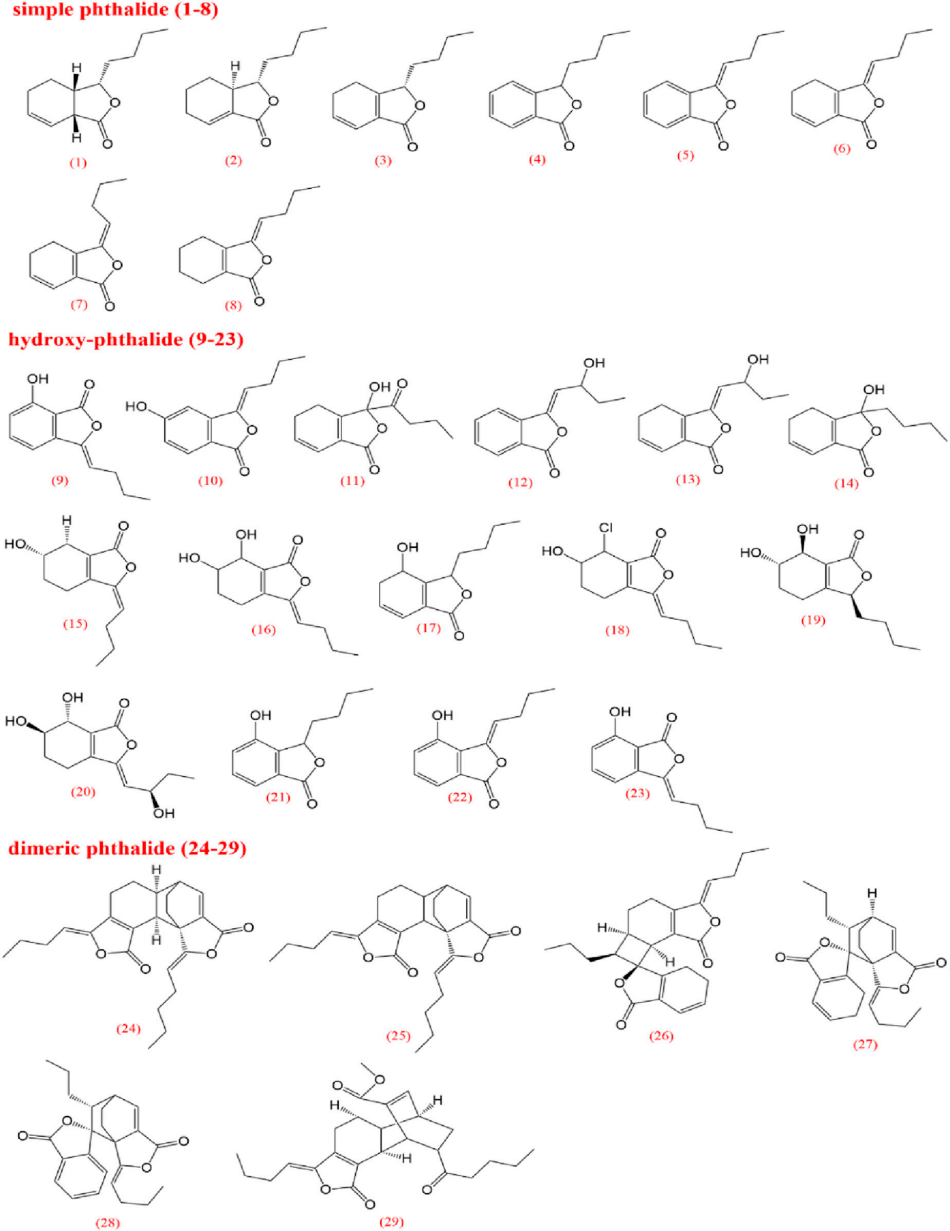
Pathalides from *Ligusticum chuanxiong*.

**FIGURE 5:**
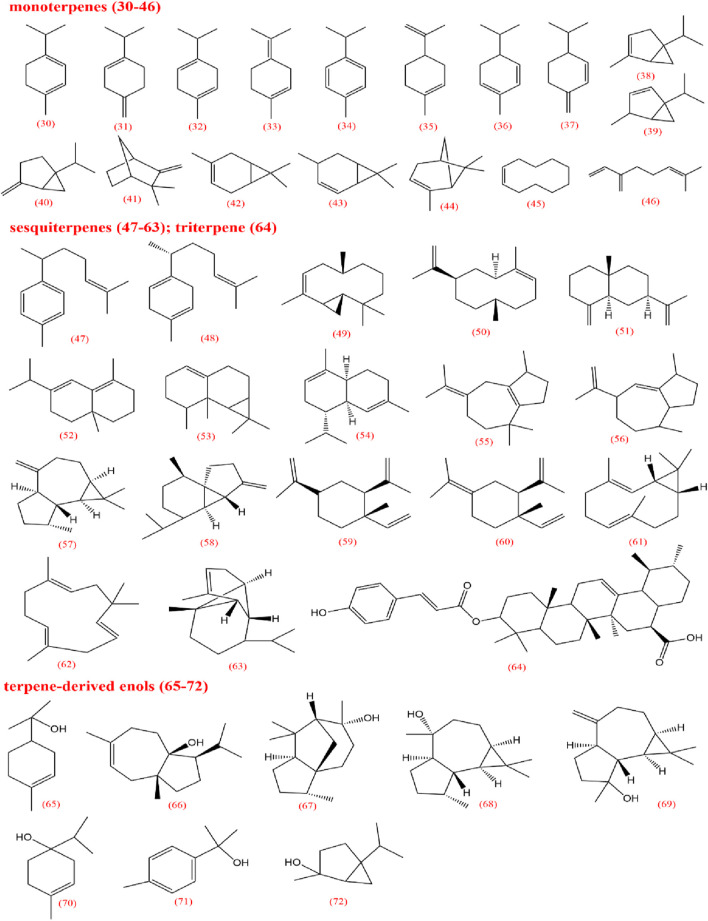
Terpenes and its enols from *Ligusticum chuanxiong*.

**FIGURE 6:**
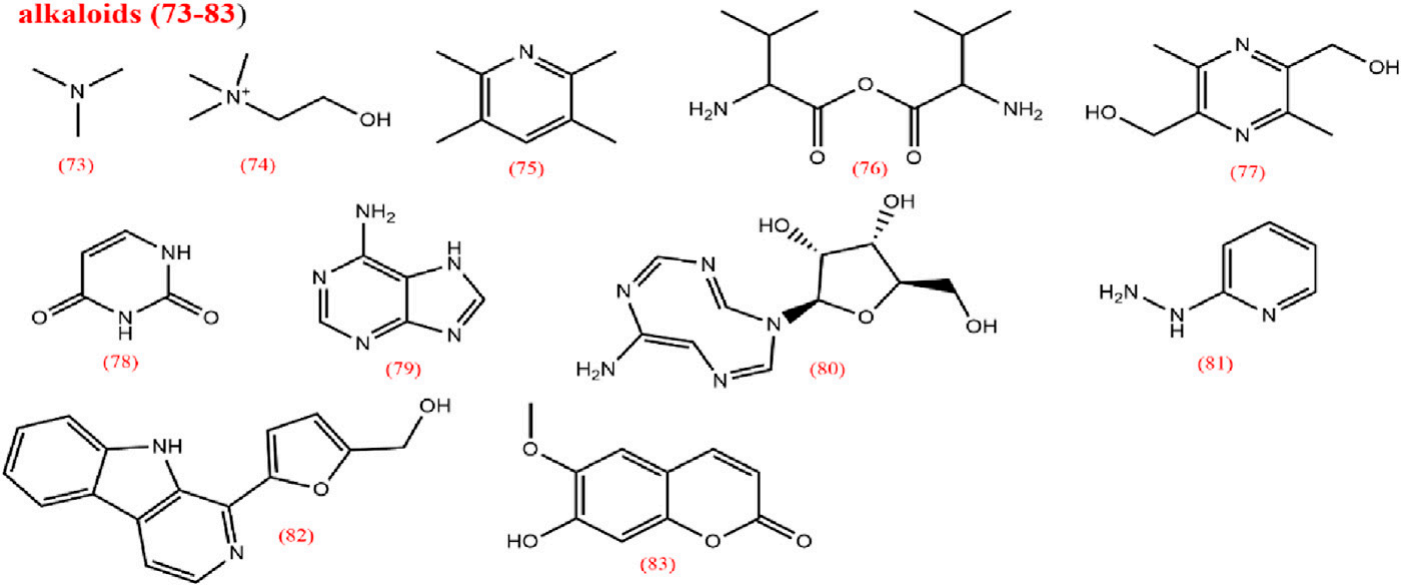
Alkaloids from *Ligusticum chuanxiong*.

**FIGURE 7:**
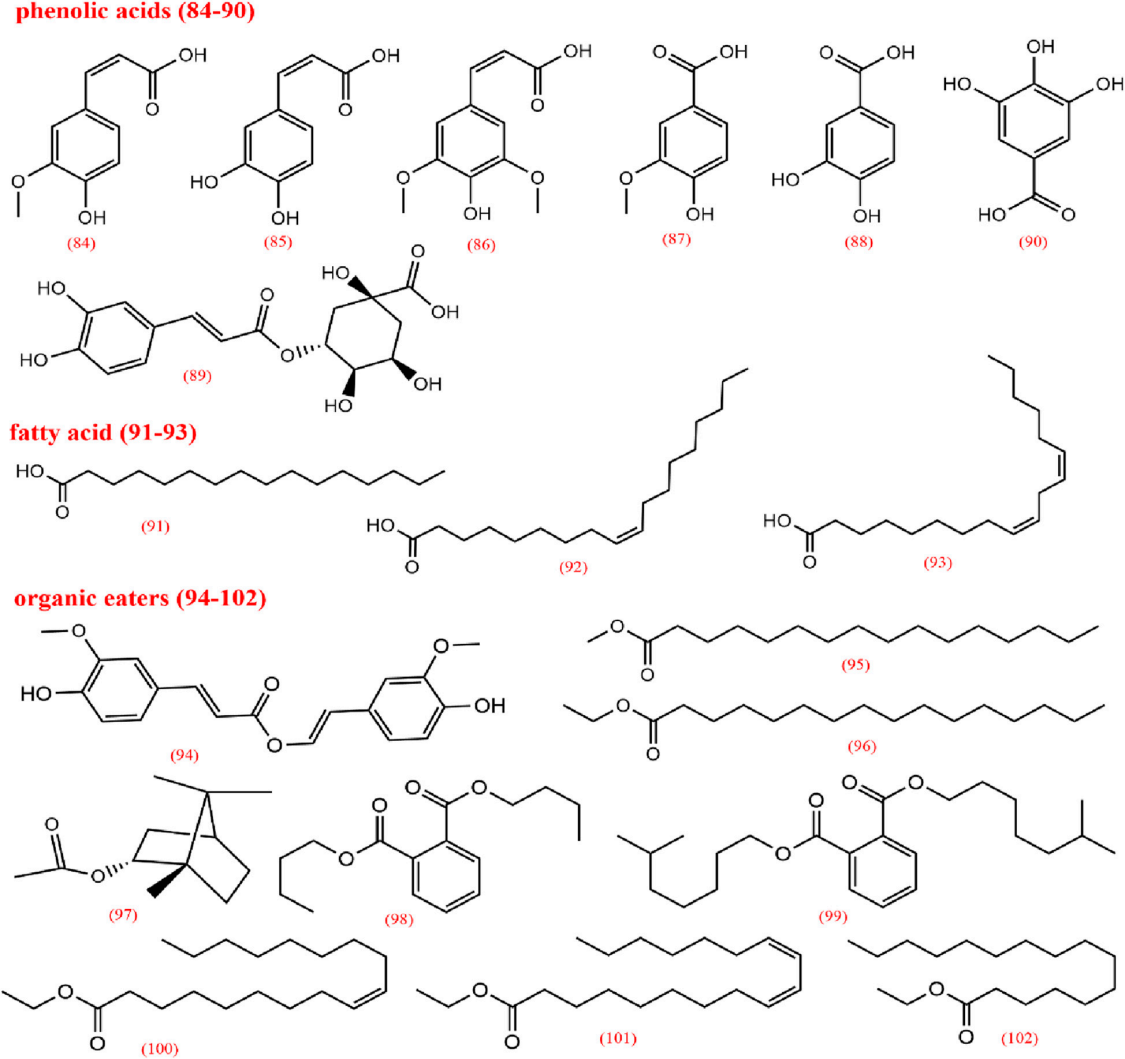
Organic acids and its ester derives from *Ligusticum chuanxiong*.

**FIGURE 8:**
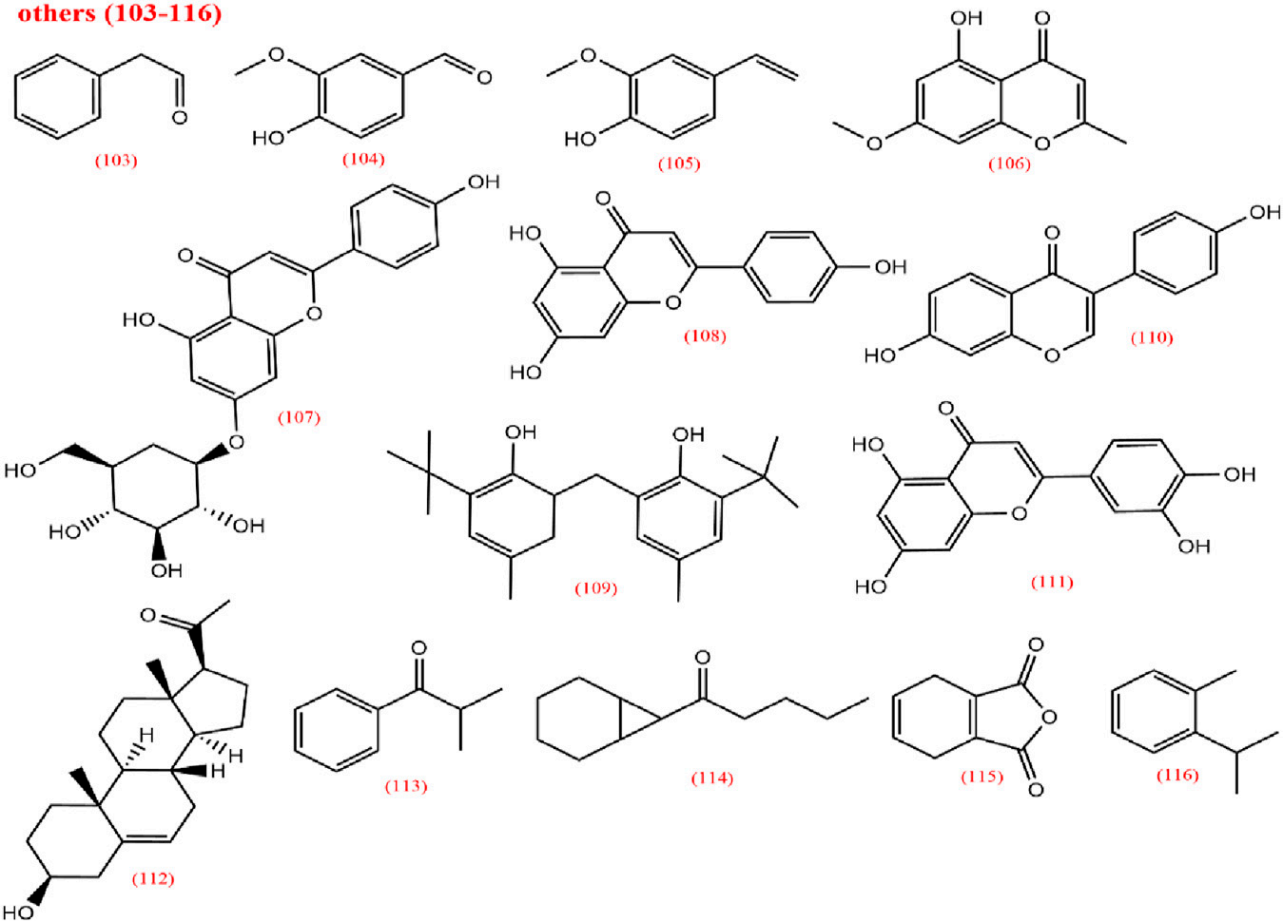
Other metabolites from *Ligusticum chuanxiong*.

To elucidate the pharmacological activities and clinical applications of CX, a comprehensive analysis of its chemical metabolites is essential. Research on the pharmacodynamic basis of CX has progressed since the 1970s, with over 100 metabolites identified to date. Key bioactive metabolites include phthalides, alkaloids, and polysaccharides. Chemical structure in SMILES format were retrieved from PubChem, and two-dimensional chemical structures representations were generated using ChemDraw Ultra 8.0. A detailed summary of the metabolites and their derivatives is presented in Table 3, with corresponding chemical structures depicted in Figures 4–8.

**TABLE 3:** Basic information of metabolites in *Ligusticum chuanxion* ***g***.

No.	Metabolite	Chemical formula	References	No.	Metabolite	Chemical formula	References
Pathalides				Terpenes and its enols			
1	cnidilide	C_12_H_18_O_2_	Li et al. (2003)	30	α-terpinene	C_10_H_16_	Shuai et al. (2022)
2	neocindilide	C_12_H_18_O_2_		31	β-terpinene	C_10_H_16_	
3	senkyunolide a	C_12_H_16_O_2_		32	γ-terpinene	C_10_H_16_	Wang et al. (2010)
4	3-butylphthalide	C_12_H_14_O_2_		33	α-terpinolene	C_10_H_16_	Yuan et al. (2000)
5	butylidene phthalide	C_12_H_12_O_2_	Wang et al. (2010)	34	p-cymene	C_10_H_14_	Wang et al. (2010)
6	z-ligustilide	C_12_H_14_O_2_	Li et al. (2003)	35	limonene	C_10_H_16_	Huang and Pu (1990)
7	e-ligustilide	C_12_H_14_O_2_	Tang et al. (2010)	36	α-phellandrene	C_10_H_16_	Siqueira et al. (2016)
8	sedanonic acid lactone	C_12_H_16_O_2_	Zhang et al. (2020b)	37	β-phellandrene	C_10_H_16_	Cheng et al. (2017)
9	senkyunolide b	C_12_H_12_O_3_	Tan et al. (2023)	38	α-thujene	C_10_H_16_	Wu and Yang (2008)
10	senkyunolide c	C_12_H_12_O_3_	Kobayashi and Mitsuhashi (1987)	39	β-thujene	C_10_H_16_	
11	senkyunolide d	C_12_H_14_O_4_	Zuo et al. (2011)	40	sabinene	C_10_H_16_	Huang and Pu (1988)
12	senkyunolide e	C_12_H_12_O_3_	Takashi et al. (1992)	41	camphene	C_10_H_16_	Zhang et al. (2024a)
13	senkyunolide f	C_12_H_14_O_3_	Li et al. (2003)	42	3-carene	C_10_H_16_	Asgary et al. (2013)
14	senkyunolide g	C_12_H_16_O_3_	Liu et al. (2016b)	43	4-carene	C_10_H_16_	Chruszcz-Lipska (2022)
15	senkyunolide h	C_12_H_16_O_3_	Li et al. (2003)	44	α-pinene	C_10_H_16_	Wang et al. (2010)
16	senkyunolide i	C_12_H_16_O_4_		45	cis-cyclodecene	C_10_H_18_	Wu and Yang (2008)
17	senkyunolide k	C_12_H_16_O_3_	Kobayashi and Mitsuhashi (1987)	46	myrcene	C_10_H_16_	Islam et al. (2020)
18	senkyunolide l	C_12_H_15_CLO_3_		47	α-curcumene	C_15_H_22_	Chen et al. (2004)
19	senkyunolide n	C_12_H_18_O_4_	Takashi et al. (1992)	48	β-curcumene	C_15_H_24_	Liu et al. (2014)
20	senkyunolide s	C_12_H_16_O_5_	Naito et al. (1996)	49	thujopsene	C_15_H_26_	Jeong et al. (2014)
21	3-butyl-4-hydroxyphthalide	C_12_H_14_O_3_	Industry (1979)	50	α-selinene	C_15_H_26_	Chen et al. (2003)
				51	β-selinene	C_15_H_24_	Wu and Yang (2008)
22	3-butylidene-4-hydroxyphthalide	C_12_H_12_O_3_	Li et al. (2002)	52	delta-selinene	C_15_H_24_	Liu et al. (2014)
				53	calarene	C_15_H_24_	Wu and Yang (2008)
23	3-butylidene-7-hydroxyphthalide	C_12_H_12_O_3_	Liu et al. (2025)	54	muurolene	C_15_H_24_	Liu et al. (2014)
				55	guaiene	C_16_H_26_	Yuan et al. (2000)
24	levistolide a	C_25_H_30_O_4_	Li et al. (2003)	56	γ-gurjunene	C_15_H_24_	Wu and Yang (2008)
25	senkyunolide p	C_25_H_30_O_4_		57	(+)-aromadendrene	C_15_H_24_	Yan et al. (2024)
26	riligustilide	C_24_H_28_O_4_		58	β-cubebene	C_15_H_24_	Yuan et al. (2000)
27	tokinolide b	C_24_H_28_O_4_		59	β-elemene	C_15_H_24_	Wang et al. (2010)
28	ansaspirolide	C_24_H_26_O_4_	Deng et al. (2006)	60	γ-elemene	C_15_H_24_	Liu et al. (2014)
29	wallichilide	C_25_H_32_O_5_	Cui et al. (2024)	61	bicyclogermacrene	C_15_H_24_	(Wang et al., 2010)
Alkaloids				62	humulene	C_15_H_24_	Yeo et al. (2021)
73	trimethylamine	C_3_H_9_NO	Zhang et al. (2021)	63	copaene	C_15_H_24_	Türkez et al. (2014)
74	choline	C_5_H_14_NO^+^	Xie et al. (2019)	64	xiongterpene	C_39_H_54_O_5_	Chang et al. (2007)
75	tetramethylpyrazine	C_9_H_13_N	Lin et al. (2022)	65	terpineol	C_10_H_18_O	Wu and Yang (2008)
76	l-valine-l-valine anhydride	C_10_H_20_N_2_O_3_	He et al. (2020)	66	carotol	C_15_H_26_O	Wang et al. (2010)
				67	cedar camphor	C_15_H_26_O	Liu et al. (2014)
77	liguzinediol	C_8_H_12_N_2_O_2_	Li et al. (2014b)	68	(−)-globulol	C_15_H_26_O	
78	uracil	C_4_H_4_N_2_O_2_	Cao et al. (1983)	69	spathulenol	C_15_H_24_O	Suručić et al. (2017)
79	adenine	C_5_H_5_N_5_	Wang et al. (1985)	70	p-menth-1-en-4-ol	C_10_H_18_O	Ruan et al. (2003)
80	adenosine	C_10_H_15_N_5_O_4_		71	p-cymen-8-ol	C_10_H_14_O	Wu and Yang (2008)
81	2-hydrazinopyridine	C_5_H_7_N_3_	Zhu and Sheng (2013)	72	cis-sabinenehydrate	C_10_H_18_O	Ruan et al. (2003)
82	perlolyrine	C_16_H_12_N_2_O_2_	Tang et al. (2000)	Aldehyde			
83	scopoletin	C_10_H_8_O_4_	(Ren et al., 2007)	103	benzeneacetaldehyde	C_8_H_8_O	Wang et al. (2010)
Organic Acids and Its Esters				Phenols			
84	ferulic acid	C_10_H_10_O_4_	Li et al. (2003)	104	vanillin	C_8_H_8_O_3_	Li et al. (2003)
85	caffeic acid	C_9_H_8_O_4_	Gang (2018)	105	p-vinylguaiacol	C_9_H_10_O_2_	Liu et al. (2014)
86	sinapic acid	C_11_H_12_O_5_	Yoon et al. (2007)	106	eugenin	C_11_H_10_O_4_	
87	vanillic acid	C_8_H_8_O_4_	Yao et al. (2020)	107	cosmosiin	C_22_H_22_O_9_	Ren et al. (2007)
88	protocatechuic acid	C_7_H_6_O_4_	Bai et al. (2021)	108	apigenin	C_15_H_10_O_5_	
89	chlorogenic acid	C_16_H_18_O_9_	Shi et al. (2024b)	109	antioxidant 2,246	C_23_H_34_O_2_	Liu et al. (2014)
90	gallic acid	C_7_H_6_O_5_	Jin et al. (2018)	Ketones			
91	palmitic acid	C_16_H_32_O_2_	International (2024)	110	daidzein	C_15_H_10_O_4_	Zhai et al. (2020)
92	oleic acid	C_18_H_34_O_2_	Cao et al. (2024)	111	meletin	C_15_H_10_O_6_	Ren et al. (2008)
93	linoleic acid	C_18_H_32_O_2_	Whelan (2008)	112	pregnenolone	C_21_H_32_O_2_	Chang et al. (2007)
94	coniferyl ferulate	C_19_H_18_O_6_	Li et al. (2003)	113	isopropyl phenyl ketone	C_10_H_12_O	Ruan et al. (2003)
95	methyl palmitate	C_17_H_34_O_2_	Sharawy et al. (2013)	114	1-bicyclo [ 4.1.0] hept-7-yl-1-pentanone	C_12_H_20_O	Wu and Yang (2008)
96	ethyl palmitate	C_18_H_36_O_2_	Ruan et al. (2003)				
97	bornyl acetate	C_12_H_20_O_2_	Yang et al. (2018)	Anhydride			
98	dibutyl phthalate	C_16_H_22_O_4_	Jang et al. (2025)	115	1,4-cyclohexadiene-1,2-dicarboxylic anhydride	C_8_H_6_O_3_	Ruan et al. (2003)
99	diisooctyl phthalate	C_24_H_38_O_4_	Luo et al. (2025)				
100	ethyl oleate	C_20_H_38_O_2_	Srimagal et al. (2017)	Aromatic Hydrocarbon			
101	ethyl linoleate	C_20_H_36_O_2_	Charakida et al. (2007)	116	1-methyl-2-isopropylbenzene	C_10_H_14_	Liu et al. (2014)
102	ethyl palmitate	C_18_H_36_O_2_	Wu and Yang (2008)				

### 2.1 Volatile oils

Volatile oils are characteristic metabolites of plants in the *Apiaceae* family, contributing significantly to the aroma of CX. Low-polarity metabolites, such as pathalides, terpenes, and their enol derivatives are typically volatile and are often present in volatile oils (Tang et al., 2022). Currently, several phthalide metabolites have been identified and isolated, which can be categorized into three main types based on their core structures: monomeric phthalides (1–8), hydroxylated phthalides (9–23), and dimeric phthalides (24–29). Representative metabolites include cnidilide, senkyunolide A, and ligustilide. Previous studies have demonstrated the efficacy of phthalides in improving blood circulation, reducing blood stasis, and providing progressive stimulation, particularly for cardiovascular and cerebrovascular diseases, as well as dysmenorrhea (Chen et al., 2011; Li J.-h. et al., 2015; Li et al., 2017). Research on terpenes remains limited; however, terpenes can be classified into monoterpenes (30–46), sesquiterpenes (47–63), triterpenes (64), and terpene derivatives (65–72).

### 2.2 Organic acids

Organic acids are primarily found in the volatile oil of CX and to a lesser extent in CX extracts, with 19 types identified. These are categorized into phenolic acids (84–90), fatty acids (91–93), and organic esters (94–102), among which ferulic acid and caffeic acid have been extensively studied. Ferulic acid, one of the most active metabolites in CX, has clinical applications, demonstrating anti-inflammatory properties, enhancement of blood flow, inhibition of platelet aggregation, prevention of thrombosis, and neuroprotective effects (Mathew and Abraham, 2004; Lin et al., 2009; Luo et al., 2019). Ferulic acid has shown antioxidant properties through its interaction with 1,1-diphenyl-2-picryl-hydrazyl radical, acting as a key antioxidant metabolite in CX (Wang et al., 2015). Meanwhile, due to its abundance, ferulic acid is frequently used as a quality control marker for CX. According to the 2020 edition of the Chinese Pharmacopoeia, the ferulic acid content in dried CX should not be less than 0.01% (Commission, 2020).

### 2.3 Alkaloids

Alkaloids are among the most important metabolites of CX; yet only a limited number of alkaloids have been isolated from CX, including organic amines (73,74), pyrazines (75–77), pyrimidines (78), purines (79,80), pyridines (81), carbolines (82), and scopolamine types (83) (Pu et al., 2019a). Contemporary research has confirmed the various pharmacological activities of alkaloids (Li et al., 2022b). In 1977, Chinese researchers identified ligustilide, also known as TMP, a pyrazine metabolite widely present in *Ligusticum* species. TMP is a representative alkaloid in CX (Chen et al., 2018; Yue et al., 2024).

### 2.4 Others

LCPs are another active metabolite of CX with multiple bioactivities, including antioxidant, immunomodulatory, antitumor, and antibacterial effects. In addition to these metabolites, CX also contains aldehydes (103), phenols (104–109), ketones (110–114), anhydrides (115), and aromatic hydrocarbons (116). This study collects and summarizes pharmacological studies of CX extracts from the past 5 years in Table 4.

**TABLE 4:** Pharmacological studies of *Ligusticum chuanxiong* extracts in the last five years.

Extracts	Metabolite	Type of study	Model	Dose range administered	Duration	Positive control	Negative control	Targets/singal pathway	Action/Disease	References
Volatile oil										
Methanol, water	Various metabolites	*In* *vivo*	-	0.02–0.1 mg/mL 25, 50, 100 mg/mL	-	Nifedipine, ferulic acid, vitamin C	Blank control group	-	Antioxidant, vasodilator, anticoagulant	Yan et al. (2022)
Ether	CX Essential Oils	*In vivo*	Cerebral Ischemia Reperfusion Injury Mouse Model	50, 60, 100 mg/kg	7 days, 15 days, 25 days	1% Sodium deoxycholate	Sham operation group, model group	-	Antioxidant, cerebral ischemia-reperfusion injury	Long et al. (2023)
Ethanol	Phthalides	*In vivo* and *in vitro*	Diabetic nephropathy model in mice	10, 20, 100 mg/kg	14 weeks	Captopril	Blank control group	Nrf2 ↑	Diabetic nephropathy	Qi et al. (2024)
Ethanol, water, etc	Chuanxiongdiolide R4、Chuanxiongdiolide R5, Chuanxiongdiolide R6, etc	*In vivo*	-	3.3, 16.8, 7.5, 12 µM	-	Nifedipine	Blank control group	Cav1.2 ↓	Vasodilation	Tang et al. (2021)
Ethanoland water	Various metabolites	*In vivo*	Mouse model of liver fibrosis	10, 17, 18, 50 mg/kg	7 days	-	Sham operation group	CTCF - c - MYC - H19 ↓	Liver fibrosis	Li et al. (2024b)
Water	CX volatile oil	*In vivo*	-	0.5%, 1%, 2%, 3%, 4%	1 h, 2 h, 4 h, 6 h, 8 h, 10 h	-	Blank control group	-	Good transdermal permeability	Hongmei et al. (2024)
Water	Various metabolites	*In vivo*	Myocardial ischemic injury model in rats	74, 149, 297 mg/kg	2 weeks	Nitroglycerin	Blank control group	ADRA1A, CHRM5 protein ↑	angina pectoris	Ming et al. (2024)
-	Essential oil of CX	*In vitro* and *in vivo*	-	1.155, 5.75, 11.55, 115.5, 1,150 μg/mL	24 h	-	Blank control group	-	Promote the transdermal absorption and reduce the toxicity of triptolide	Cheng et al. (2024)
-	Essential oil of CX	*In vitro*	Common carotid artery thrombosis rats, tail thrombosis rats, platelet aggregation rat model	0.5, 1.5, 4.5 g/kg 0.4, 1.6, 6.4 g/kg 0.6, 1.8, 5.6 g/kg	3.5 days	Aspirin	Blank control group	COX-1 ↓	Prevent arterial thrombosis	Lin et al. (2024)
Water vapor	Essential oil of CX	*In vitro*	Amigraine rats	45, 90, 135 mg/kg	7 days	Zolmitriptemp	Blank control group	5-HT, 5-HT1 Bprotein ↑	Relieve and treat migraines	Tianhao et al. (2022)
-	Dl-3-n-butylphthalide (NBP)	*In vivo*	traumatic brain injury rats	10, 20, 40 mg/kg	7 days	-	Blank control group	lRE1/XBP1 ↓	Improvement neurologieal function	Yun et al. (2021)
-	Essential oil of CX	*In vitro*	-	20, 40, 80 μmol/L	24 h	-	Blank control group	EGFR/VEGF-A ↓	glioma angiogenesis	Xuhui et al. (2023)
Water vapor	Essential oil of CX	*In vitro*	-	1.56 × 10^^−3^, 6.25 × 10^^−3^ μL/mL	48 h	-	Blank control group	LRIG2, EGFR, VEGF-A ↓	Inhibit glioma and angiogenesis	Yumei et al. (2022)
Water vapor	Essential oil of CX	*In vitro*	-	1.25, 2.5, 5, 10, 20, 40, 60, 80 μg/mL	12 h	-	Blank control group	IL-6 ↓	Anti-inflammatory	Yiyu et al. (2023)
-	Ligustilide、Senkyunolide A、Senkyunolide I	*In* *vitro*	-	20, 40, 80 μg/mL	-	-	Blank control group	P-glycoprotein, claudin-5, ZO-1 ↓	Inhibit damage cells and promote the transmembrane transport	Shanshan et al. (2021)
Organic acids										
95%ethanol	Ferulic acid	*In vivo* and *in vitro*	-	5.8 g/kg 1, 10, 100 μmol/L	0.5 h, 1 h, 2 h, 4 h, 6 h, 8 h	-	Blank control group	NF-Kb/MAPK/c-Jun/TLR4 ↓	Diseases of the central nervous system	Mingyan et al. (2024)
water	Ferulic acid	*In vivo* and *in vitro*	Cholestatic liver injury mice	50 mg/kg 80 μg/mL	4 days; 24 h	Nimodipine group	Blank control group, model group	HSCs activation ↓	Cholestatic liver injury, anti-inflammatory, antioxidant	Yajing et al. (2023)
75% ethanol	Isogreen source acid A	*In vitro*	-	1.5 mg/L	60 s	Agaqu team	Blank control group	factor Xa ↓	Thromboembolic disease	Yang et al. (2020)
ethyl acetate	Phthalate derivatives	*In* *vitro*	-	500.00 μM	3 min	Allopurinol group	Blank control group	Xanthine oxidase ↓	Anti-gout	ManLi et al. (2023)
Alkaloids										
80% ethanol	Ligustrazine	*In vivo*	Mouse model of hepatic fibrosis	10 mg/kg	2 days, 4 days	-	Blank control group	CF-c -MYC-H19, cholangiocyte ↓	Liver fibrosis	Li et al. (2024b), Li et al. (2024c)
Water	Adenines	*In vitro*	-	12.5, 25, 50 μM	24 h	Curcumin group	Blank control group	TNF-α, IL-6 ↓	Anti-inflammatory	Feng et al. (2023a)
Water	Isoindoline alkaloids	*In vitro*	-	1, 10, 50 μM	10 min	Verapamil hydrochloride group	Blank control group	inward flow of extracellular calcium ions↓	Relaxation of uterine smooth muscle	Juan et al. (2022)
80% ethanol	Total alkaloids	*In vivo*	Migraine Rat Model/Migraine Mouse Model	12.5, 25, 50 mg/kg	1 week; 3 days	Zolmitriptan group	Blank control group, model group	5-HT ↑; c-Jun ↓	migraine	Zhong-Hui et al. (2019)
Other										
Water	Polysaccharide (LCP70-2 A)	*In vivo* and *in vitro*	Zebrafish embryo model	50, 100, 200 μg/mL	24 h, 3 days	LPS group	Blank control group	immune cells, NO, TNF-α, IL-6, IL-1β ↑	Anti-inflammatory	Shaojie et al. (2021)
80% ethanol	Polysaccharide (LCX0、LCX1、LCX2)	*In vitro*	-	0.57, 2.57.2.95, 8 μg/mL 25–1,000 μg/mL	48 h	-	Blank control group	-	Antioxidant/anti-cancer	Hu et al. (2016b)

-, unavailable data; ↑, Upward adjustment, promotion or increase; ↓, Downward, inhibit or decrease c-Jun, c-Jun N-terminal kinase; IL, interleukin; MAPK, mitogen-activated protein kinase; Nrf2, Nuclear factor erythroid 2-related factor 2; NF-κB, nuclear factor kappa-B; TLR4, Toll-like receptor 4; 5-HT, 5-hydroxytryptamine; TNF, tumor necrosis factor; NO, nitric oxide; ZO-1, Zonula occludens-1; LRIG2, Leucine Rich Repeats And Immunoglobulin Like Domains 2; EGFR, epidermal growth factor receptor; VEGF-A, Vascular endothelial growth factor-A; HSC, hepatic stellate cell and XBP1, X-Box Binding Protein 1.

## 3 Pharmacological properties of *Ligusticum chuanxiong*

### 3.1 Central nervous system

#### 3.1.1 Cerebral ischemia

Cerebral ischemia/reperfusion (I/R) injury is commonly associated with high rates of disability and mortality due to its complex pathological mechanisms. CX, a traditional medicine, is widely used in the treatment of ischemic stroke. Several metabolites of CX have demonstrated efficacy in managing cerebral ischemia by modulating oxidative stress, protecting blood vessels, and regulating inflammation. For instance, the National Medical Products Administration (NMPA) recognized butylphthalide as a therapeutic agent for ischemic stroke in 2005 (Zou et al., 2018). TMP inhibits platelet adhesion and the release of inflammatory factors by suppressing the P38 mitogen-activated protein kinase (MAPK) and nuclear factor kappa-B (NF-κB) signaling pathways, thereby protecting microvascular endothelial cells in the brain (Zhang H. et al., 2020). Ligustilide activates adenosine 5‘-monophosphate (AMP)-activated protein kinase (AMPK) signaling and PTEN induced putative kinase 1 (PINK1)/Parkin expression, which helps alleviate ischemic stroke damage (Mao et al., 2022; Wu et al., 2022). In a mouse model of induced intracerebral hemorrhage, ligustilide and senkyunolide H were shown to exert potent neuroprotective effects against hemorrhagic stroke by inhibiting the Paired Related Homeobox 1 (Prx1)/Toll-like receptor 4 (TLR4)/NF-κB pathway, and subsequent immune and neuroinflammatory damage (Han et al., 2018). In a subacute ischemic rat model, TMP was found to inhibit the overexpression of aquaporin 4 (AQP4) and connexin 43 (Cx43) in astrocytes by altering A1-A2 reactivity. TMP also activates the fibroblast growth factor (FGF) 2/phosphoinositide 3-kinase (PI3K)/Akt pathway, which may contribute to its promotion of neurovascular repair following ischemic stroke (Feng X. F. et al., 2023). Additional studies have reported that CX and Binpian *[Cinnamomum camphora(L.)Presl]* exhibit significant synergistic effects in promoting neurogenesis and reducing astrocyte generation, with CX playing a central role and Binpian enhancing its effects. Binpian is frequently combined with CX to improve the treatment of ischemic stroke (Song et al., 2023; Zhang X. et al., 2024).

#### 3.1.2 Inflammation inhibition

Senkyunolide A and Z-ligustilide, two isolated metabolites, demonstrate inhibitory effects on neuroinflammation (Or et al., 2011). In microsphere-embolized (ME) rats, CX promotes neurogenesis and reduces inflammatory factors, thereby protecting neurons from cerebral ischemia (Wang et al., 2020). TMP has been shown to decrease the levels of inflammatory factors, such as tumor necrosis factor (TNF)-α, interleukin (IL)-1β, IL-6, and matrix metalloproteinase (MMP)-9, in ischemic brain tissue, exerting neuroprotective effects in mice subjected to I/R injury. This neuroprotective effect may be mediated by the upregulation of monocyte chemoattractant protein-induced protein 1 (MCPIP1), as the neuroprotection and anti-inflammatory effect were inhibited upon knockdown of MCPIP1 using specific small interfering RNA (siRNA) (Jin et al., 2021). TMP also protects retinal ganglion cells by inhibiting apoptosis and autophagy via the PI3K/Akt/mammalian target of rapamycin (mTOR) pathway. Additionally, Senkyunolide I has been found to protect mouse neuroblastoma cells from glutamate-induced injury by downregulating the c-Jun N-terminal kinase (c-JUN)/cysteinyl aspartate specific proteinase 3 (caspase-3) pathway and attenuating apoptosis (Wang M. et al., 2021). Furthermore, ligustilide exerts protective effects against oxygen-glucose deprivation/reperfusion (OGD/R) injury in PC12 cells by inhibiting apoptosis through the mitochondrial pathway and promoting autophagy via the Liver kinase B1 (LKB1)-AMPK-mTOR signaling pathway (Zhao et al., 2020). Ma et al. (2024) found that QBT, a novel phthalein metabolite, significantly reduced cognitive dysfunction and neuronal damage in rats with vascular dementia (VaD). It also inhibited VaD-induced hyperactivation of microglia and astrocytes, along with associated inflammatory responses. Moreover, QBT exerts anti-inflammatory effects by inhibiting the chemokine C-X-C motif ligand 12 (CXCL12)/chemokine C-X-C motif receptor 4 (CXCR4) axis and its downstream Janus kinase 2 (JAK2)/signal transducer and activator of transcription 3 (STAT3) and PI3K/Akt/NF-κB pathways, thereby attenuating neuroinflammatory responses both *in vivo* and *in vitro*.

#### 3.1.3 Alzheimer’s disease

Alzheimer’s disease (AD) is the most common neurodegenerative disorder, leading to various functional impairments, including memory loss, dementia, and aphasia. It is the third leading cause of disability and death in the elderly, following cardiovascular diseases and malignant tumors, and is considered a major societal burden (Jeremic et al., 2021). Several hypotheses have been proposed regarding the pathogenesis of AD, with the most widely accepted mechanisms involving the excessive accumulation of beta-amyloid (Aβ) outside neurons and the aggregation of hyperphosphorylated tau protein within neurons, resulting in the formation of neurofibrillary tangles (NFTs) (Du et al., 2018; Scheltens et al., 2021). Despite extensive efforts to develop therapeutic strategies for AD, only a few drugs have been approved for treatment, all of which merely provide symptomatic relief and are associated with significant side effects. Effective cures or preventive treatments remain scarce.

In the context of tau pathology, CX has been reported to target 70 key pathways associated with tau-related pathological mechanisms, including interactions with astrocytes, endothelial cells, and microglia. This action effectively reverses tau hyperphosphorylation and promotes its degradation through the lysosomal pathway, thereby mitigating tau-mediated intracellular transport impairment, axonal and synaptic damage, and neuronal death. The primary active metabolites identified in this process include neocnidilide, ferulic acid, Z-ligustilide, and butylidene phthalide, which have demonstrated neuroprotective effects against AD. In contrast, TMP, a widely used therapeutic agent for I/R-induced brain injury, has been associated with only two tau-related targets, suggesting limited therapeutic relevance in tau pathology-based AD (Zeng et al., 2022). However, compared to the control group, the TMP-related group exhibited significant improvements in learning and memory abilities in transgenic AD mice, along with reduced levels of Aβ and tau phosphorylation in the brain (Huang et al., 2021). These effects may be attributed to TMP’s ability to regulate mitochondrial function, maintain synaptic integrity and cytoskeletal stability, and modulate proteins associated with adenosine triphosphate (ATP) and guanosine triphosphate (GTP) binding. Another potential mechanism underlying TMP’s anti-AD’s effects involves the downregulation of Cullin 4B (CUL4B) in the brain tissue of AD mice, which inhibits the ubiquitination of somatostatin receptor 4 (SSRT4) and upregulates SSRT4 protein levels. This mechanism has been confirmed in mice with CUL4B overexpression or SSRT4 silencing (Weng et al., 2021). Additionally, TMP may partially restore cholinergic neuronal function in streptozotocin (STZ)-induced AD rats by inhibiting glycogen synthase kinase (GSK)-3β activity, thereby improving learning and memory deficits (Lu F. et al., 2017).

One study demonstrated that QBT exhibits strong biological activity against cerebrovascular diseases. In VaD rats, QBT reversed aberrant changes associated with ferroptosis and pyroptosis by upregulating the nuclear factor erythroid 2-related factor 2 (Nrf2)/cystine-glutamate antiporter (xCT)/glutathione peroxidase 4 (GPX4) pathway, downregulating the nucleotide-binding oligomerization domain-like receptor family pyrin domain-containing 3 (NLRP3)/cysteine-requiring aspartate protease-1 (Caspase-1)/gasdermin D (GSDMD) pathway, and inhibiting both ferroptosis and pyroptosis in neuronal cells. These actions exerted neuroprotective effects, significantly reducing neuronal damage and cognitive dysfunction in VaD rats (Feng et al., 2024). Furthermore, the methanol extract of CX effectively mitigated cognitive impairment caused by bilateral common carotid artery stenosis, primarily through the inhibition of astrocytes and microglia (Lim et al., 2024).

#### 3.1.4 Others

Parkinson’s disease (PD) is a progressive neurodegenerative disorder characterized by a gradual loss of motor function, with the primary pathological hallmarks being the sustained degeneration of dopaminergic neurons in the substantia nigra and the intracellular accumulation of α-synuclein in the form of Lewy bodies and Lewy neurites (Kouli et al., 2018). CX may play a role in PD biology, as 53 metabolites in CX have been found to affect the biological compartments of human neuronal oxidative stress (hNOS) cells derived from PD patients. These metabolites may interfere with the biological functions of cellular organelles, including early endosomes, lysosomes, and autophagosomes. Additionally, TMP downregulates neuroinflammation markers such as NF-κB, inducible nitric oxide synthase (iNOS), and cyclooxygenase-2 (COX2) (Michel et al., 2017). Canhui and Mengchan (2023) found that ligustilide improved the survival rate of PD cell models, reduced cell cycle arrest in the G0/G1 phase, and inhibited apoptosis, primarily by suppressing the activation of the p38 MAPK/c-JUN signaling pathway.

The antimigraine effects of total alkaloids in CX have been confirmed to be mediated by the upregulation of 5-hydroxytryptamine (5-HT) and its metabolite 5-hydroxyindole acetic acid (5-HIAA), achieved through increased expression of the 5-Hydroxytryptamine Receptor 1B (5-HT1B) and suppression of c-Jun (Pu et al., 2019b). Furthermore, TMP selectively inhibits c-Jun phosphorylation and reduces the expression of MMP-2/9 and pro-inflammatory cytokines secreted by astrocytes, thereby alleviating neuropathic pain caused by chronic compression injury (CCI). TMP also inhibits the activation of the NF-κB pathway in microglia by upregulating the expression of silent information regulator 1 (SIRT-1), thereby reducing neuroinflammatory responses and alleviating migraine (Chen et al., 2024).

Huntington’s disease (HD) is a neurodegenerative disorder with an autosomal dominant inheritance pattern, primarily characterized by motor dysfunction, ataxia, and cognitive decline. 3-Nitropropionic acid (3-NP) is commonly used to induce HD-like symptoms in animal models. Treatment with TMP significantly improves behavioral outcomes in 3-NP-induced HD rats, with biochemical analyses indicating a marked reduction in oxidative stress parameters (Danduga et al., 2018).

### 3.2 Cadio-vascular system

#### 3.2.1 Myocardial ischemia

Myocardial ischemia improvement is one of the most significant pharmacological actions of CX and its most widely applied use in clinical practice. Research indicates that CX exerts anti-inflammatory, antioxidative, and anti-apoptotic effects on cardiomyocytes subjected to I/R injury, while also promoting coronary blood flow and myocardial metabolism, thereby limiting the extent of myocardial infarction (Zheng et al., 2018). Additionally, studies have shown that CX protects against acute myocardial ischemia induced by isoproterenol through activation of the PI3K/Akt/GSK-3 signaling pathway (Yang et al., 2019).

Wang et al. (2024) demonstrated that TMP intervention reduced cardiomyocyte apoptosis induced by OGD/R and I/R, enhanced cellular viability and autophagy, and improved myocardial tissue necrosis in I/R mice in a dose-dependent manner. Ligustilide has been shown to attenuate myocardial injury caused by inflammation, reduce fibrosis and hypertrophy of myocardial cells, and inhibit ventricular dilation following myocardial infarction. Furthermore, ligustilide provides protective effects in familial dilated cardiomyopathy. When combined with other Chinese herbal medicines, ligustilide can enhance therapeutic efficacy synergistically; for example, its combination with berberine has demonstrated more effective antiplatelet and anti-inflammatory effects in rats with acute myocardial infarction (Zhang et al., 2016).

In addition to inflammation and oxidative stress, autophagic dysfunction plays a significant role in various cardiovascular diseases. Studies have demonstrated that the lactone metabolite of ligustilide can activate the PI3K/Akt/mTOR signaling pathway, restore autophagic flux, and exert cardioprotective effects (Wang et al., 2018). Moreover, CX protects the myocardium from I/R injury by inhibiting Beclin-1-related autophagic dysfunction and enhancing the translocation of B-cell lymphoma-2 (Bcl-2) through increased expression of 14-3-3γ(Zuo et al., 2019).

#### 3.2.2 Atherosclerosis

AS is characterized by the accumulation of lipids, complex carbohydrates, and/or thrombus formation, accompanied by a progressive fibrotic response and calcium deposition, ultimately leading to arterial thickening, hardening, and luminal narrowing (Libby, 2021). Zhang et al. (2024b) explored the mechanisms through which Zhizi *(Gardenia jasminoides Ellis)* and CX botanical drugs attenuate the progression of AS via Deoxyribonucleic Acid (DNA) methylation. The results indicate that both Zhizi and CX effectively slow AS progression by inhibiting MAPK/extracellular signal-regulated kinase (ERK)-mediated apoptosis through hypermethylation of the fibroblast growth factor receptor 3 (FGFR3) promoter region. Additionally, they alleviate AS by inhibiting the TNF/NF-κB axis and M1 macrophage polarization (Zhang et al., 2024c). Liu et al. (2024) demonstrated for the first time the potential of oral Z-ligustilide treatment to alleviate AS by reducing inflammation and enhancing intestinal barrier function. TMP exerts therapeutic effects on AS primarily through its anti-inflammatory, antioxidant, and lipid-lowering actions, effectively inhibiting lipid accumulation and enhancing intracellular cholesterol efflux. Key targets implicated in these mechanisms include peroxisome proliferator-activated receptors (PPAR), sterol regulatory element-binding proteins (SREBPs)/cleavage activating protein (SCAP), progesterone and adiponectin receptors 3, scavenger receptors (SR-A, CD36), ATP-binding cassette transporters (ABCA1 and ABCG1), and the PI3K/Akt and p38/MAPK signaling pathways (Chen et al., 2017; Duan et al., 2017).

#### 3.2.3 Qthers

In the management of hypertension, TMP has been reported to exert dual actions: it blocks the entry of extracellular Ca^2+^ through calcium channels and inhibits the release of Ca^2+^ stored within vascular smooth muscle cells (SMCs) (Ren et al., 2012). The Kcnma1 gene encodes the large conductance calcium-activated potassium channels (BKCa), which are critical for treating various cardiovascular, muscular, and nervous system disorders. Research has shown that TMP helps maintain BKCa β1 protein expression by inhibiting endoplasmic reticulum stress, restoring BKCa currents in porcine coronary artery SMCs, and improving BKCa-mediated vasodilation in coronary arteries (Sun et al., 2019; Szteyn and Singh, 2020).

Liguzinediol exhibits a dose-dependent positive inotropic effect in doxorubicin-induced heart failure mice, indicating its potential as a treatment for heart failure without causing adverse reactions such as arrhythmias or hypotension. This beneficial effect is primarily mediated by an increase in the bcl-2/Bcl-2 associated X protein (Bax) ratio and the suppression of caspase-3 expression and NK-κB activation (Li et al., 2014a). Sodium ferulate has been shown to mitigate myocardial hypertrophy induced by abdominal aorta coarctation by inhibiting protein kinase C beta (PKC-β) and activating the MAPK signaling pathways (Luo et al., 2019).

TMP also demonstrates inhibitory effects on thrombosis. It exerts its antithrombotic effects by reducing inflammatory cytokines and adhesion molecules, modulating MAPK signaling, attenuating oxidative stress, preventing apoptosis, inhibiting platelet aggregation, and suppressing the expression of fibrinogen and von Willebrand factor (Yang et al., 2023). In addition to TMP and ferulic acid, four other effective metabolites have been identified: neochlorogenic acid, 1-H-benzimidazole-2-amine, 3,8-dihydroxyacyl lactone, and CX triterpenes. These metabolites interact with the core amino acids of target proteins, effectively inhibiting blood clot formation.

### 3.3 Effects of *Ligusticum chuanxiong* on other systems or organs

#### 3.3.1 Respiratory system

Studies have found that CX adjunctive therapy can significantly improve the clinical symptoms of patients with chronic idiopathic pulmonary fibrosis (Shupei, 2010). The use of microemulsion formulations encapsulating the volatile oils of Danggui [*Angelica sinensis (Oliv.) Diels*] and CX has been shown to enhance their ability to alleviate acute lung injury (ALI) (Zhang et al., 2022). TMP is primarily regarded as an active medicinal metabolite of CX that targets the respiratory system. *In vivo* studies have demonstrated that TMP inhibits the proliferation of airway SMCs by reducing p-ERK 1/2 levels, a process associated with the MAPK signaling pathway (Li et al., 2018). Jiang et al. confirmed that TMP exhibits both anti-inflammatory and anti-apoptotic activities in macrophages, which have been validated *in vivo* and *in vitro*. These effects involve the TLR4/Tumor necrosis factor receptor-associated factor 6 (TRAF6)/NF-κB/NLRP3/caspase-1 and TLR4/cysteine-requiring aspartate protease-8 (caspase-8)/caspase-3 signaling pathways (Jiang R. et al., 2021). Furthermore, TMP has been shown to alleviate lung inflammation in an ovalbumin-induced asthma mouse model by downregulating C-C Motif Chemokine Receptor 1 (CCR1), STAT3, and p38 MAPK proteins, thereby inhibiting airway hyperresponsiveness and the release of inflammatory and chemotactic factors (Wei et al., 2016). These findings suggest that TMP may have potential in treating ALI and acute respiratory distress syndrome (ARDS) primarily through reducing inflammatory cell infiltration and lowering pro-inflammatory factor secretion in the alveoli. Furthermore, TMP has shown efficacy in alleviating ALI caused by sepsis, reducing inflammatory cell infiltration, and mitigating lipopolysaccharide-induced lung tissue damage. TMP significantly inhibits the Rac Family Small GTPase 1 (Rac1)/LIM Domain Kinase 1 (LIMK1)/Zonula occludens-1 (ZO-1)/occludin signaling pathway (Min et al., 2022).

#### 3.3.2 Hepatic protection

Ferulic acid demonstrates anti-hepatic stellate cell (HSC) activity by inhibiting focal adhesion kinase (FAK) and blocking the Smad and ERK1/2 signaling pathways, highlighting its potential in treating liver fibrosis (Xu et al., 2015). In a rat model of thioacetamide-induced liver fibrosis, butylidene phthalide exhibited significant anti-fibrotic effects by inhibiting epithelial-mesenchymal transition, reducing inflammatory responses, and promoting hepatocyte proliferation (Chuang et al., 2016). Additionally, CX combined with Danggui has been shown to prevent liver fibrosis (Wu et al., 2021). TMP has been reported to effectively attenuate hepatic I/R injury by inhibiting neutrophil extracellular trap formation and nicotinamide adenine dinucleotide phosphate (NADPH) oxidase (Liu et al., 2021). In the study by Lu et al., TMP was also found to confer hepatoprotective effects and improve hepatic steatosis via Nrf2 activation (Lu C. et al., 2017). Furthermore, Lu et al. (2023) reported that the organic acids in CX can alleviate liver injury through multiple signaling pathways, primarily involving the positive regulation of nitric oxide (NO) biosynthesis, Toll-like receptor signaling, NOD-like receptor signaling, and TNF signaling pathways. Additionally, the phthalate ester extract from CX exhibits stronger anti-cholestatic activity, both *in vivo* and *in vitro*, compared to other CX extracts. Its protective effect on bile duct cells and HSCs is mediated by reducing the transcription and release of plasminogen activator inhibitor-1 (PAI-1) and fibronectin through lysine acetyltransferase 2A (KAT2A)/histone 3 lysine 9 acetylation (H3K9ac) (Li Y. et al., 2023).

#### 3.3.3 Nephric protection

Studies have shown that CX and Dahuang*(Rheum officinale Baill.)* may inhibit renal tubular epithelial cell apoptosis and improve acute kidney injury (AKI) and renal fibrosis by suppressing the p38 MAPK/p53 signaling pathway (Li J. et al., 2023). Xia et al. (2024) found that ligustilide alleviates oxidative stress during renal I/R injury by maintaining Sirt3-dependent mitochondrial homeostasis. Qi et al. (2024) identified potential effective metabolites of CX in renal protection, revealing that phthalides improve hyperglycemia-induced renal dysfunction by enhancing Nrf2 activation, reducing collagen deposition, and alleviating inflammation and oxidative stress. Levistolide A mitigates AKI through its antioxidant properties and modulation of the TLR4/NF-κB signaling pathway (Shi J. et al., 2024). TMP exhibits anti-apoptotic and therapeutic effects in diabetic nephropathy (DN) rats by activating the Akt signaling pathway and reducing oxidative stress (Rai et al., 2019). Its renal protective effects include attenuation of renal pathological changes, reduction in blood urea nitrogen, serum creatinine, 24-h urinary albumin, and glycated hemoglobin (HbA1c) levels, as well as an improvement in creatinine clearance (Zhuang et al., 2020). Additionally, TMP plays a significant protective role in AKI, primarily by downregulating the expression of nucleotide-binding oligomerization domain 2 (NOD2), which reduces renal cell apoptosis in a rat ischemia-reperfusion injury model (Jiang G. et al., 2021). TMP has been applied clinically to mitigate AKI. Senkyunolide I alleviates renal I/R injury by inhibiting oxidative stress, endoplasmic reticulum stress, and apoptosis (Zhu et al., 2022).

#### 3.3.4 Cytotoxic effects

Tumor metastasis plays a critical role in the progression of malignant tumors, inhibiting autophagy and apoptosis in cancer cells, thereby promoting uncontrolled cell division and proliferation. CX, a natural plant extract, contains various metabolites with multi-targeted antitumor activities, including the promotion of tumor cell apoptosis and the inhibition of metastasis (Wang K. et al., 2021). Perlolyrine has been identified as a metabolite with antiproliferative effects on human gastric cancer cell lines (Lee et al., 2016); metabolites CX0, CX1, and CX2 exhibit inhibitory effects on the growth of HepG2, SMMC7721, A549, and HCT-116 cells (Hu et al., 2016a); metabolites CXP-1a and CXP-3a stimulate macrophages to produce NO, TNF-α, IL-6, and IL-12, while inhibiting the growth of HepG2 and Hep3B cells through immune modulation by blocking the cell cycle in the G0/G1 phase, thus promoting cancer cell apoptosis (Zhong et al., 2021). Jung et al. (2025) elucidated the molecular mechanisms underlying TMP’s anticancer effects: TMP induces apoptosis in HCCLM3 and Hep3B cells by activating key apoptosis factors and inhibiting proteins associated with cell survival and angiogenesis. It enhances autophagy by promoting the formation of autophagosomes and stimulating autophagy-related proteins, while upregulating SHP-1 to inhibit the activation of the STAT3 signaling pathway, thereby suppressing tumorigenesis and activating cell death pathways. TMP significantly inhibits tumor growth and triggers apoptosis and autophagy in tumor tissues.

## 4 Clinical practice of *Ligusticum chuanxiong*

Seventeen Chinese patent medicines containing CX as the main ingredient were retrieved from the NMPA, including CX Cha Tiao Power, CX Cha Tiao Granules, CX Cha Tiao Pills, Compound CX Capsules, DaCX Tablets, CX Cha Tiao Tablets, CX Qingnao Granules, CX Cha Tiao Dai Pao Ji, CX Cha Tiao Dripping Pills, CX Cha Tiao Oral Liquid, CX Cha Tiao Chong Ji, Shiquan Dabu Pills, Compound CX Tablets, DaCX Oral Liquid, CX Cha Tiao Dai Pao Cha, and DaCX Granules.

Qunfang (2024) found that the combining of CX Cha Tiao Power and acupuncture significantly improved clinical efficacy, alleviated headache symptoms, and positively influenced prognosis. Lihua et al. (2023) study on 224 patients with chronic cerebral ischemia and headache using CX Qingnao Granules showed a significantly higher total clinical efficacy rate than the control group (P < 0.05), with reductions in headache frequency and protection of cognitive function. Ran and Qingfang (2024) used DaCX Oral Liquid to treat 192 stroke sequela patients, finding a clinical efficacy rate of 94.79% in the treatment group (DaCX Oral Liquid + conventional therapy), significantly higher than the 84.38% in the control group. Jianfang and Wenlong (2022) investigated the clinical effect of Compound CX Capsules combined with Arorol hydrochloride tablets in treating angina pectoris, reporting a significantly higher total effective rate in the treatment group (98.31% vs. 86.44%, P < 0.05). This combination therapy not only improved symptoms but also promoted cytokine level improvements and enhanced quality of life. CX-based formulations have been widely used in clinical practice for related diseases, but most research is still focused on Chinese literature, with limited studies published in English. Additionally, these studies often combine CX with other clinical treatments (e.g., acupuncture and Western medicines). The effectiveness of CX-based formulations in clinical applications still requires further validation through multicenter, large-sample studies.

## 5 Safety

As a traditional Chinese botanical drug, CX is not classified as highly toxic, moderately toxic, or low-toxicity in the 2020 edition of the Chinese Pharmacopoeia. To date, no significant organ toxicity has been reported in relation to its use, distinguishing it from other botanical drugs known to have toxic effects. For example, Baihuasheshecao (*Hedyotis diffusa Willd.)* has been linked to toxicity in the cardiac, digestive, neurological, urinary, and respiratory systems; Maqianzi *(Strychnos nux-vomica Linn.)* is toxic to the digestive, hepatic, renal, and nervous systems; and Feiyangcao*(Euphorbia hirta Linn.)* exhibits corrosive toxicity to the digestive system (Li N. et al., 2022).

In a study by Zhang et al., the acute toxicity and dermal sensitivity of essential oil (EO) extracted from CX were evaluated. The results showed that the EO caused mild irritation to rabbit skin, but no significant signs of pigmentation, bleeding, roughness, or skin thinning were observed in guinea pigs after 7 days of treatment. The median lethal doses (LD50) were determined to be 7.23 g/kg and 2.25 g/kg for oral and intraperitoneal administration, respectively, equivalent to approximately 14,606 and 5,091 times the clinical dosage (Zhang et al., 2012). Furthermore, CX demonstrated low embryotoxicity, as indicated by the median inhibitory proliferation values of mouse embryonic stem cells (9.39 mg/mL) and mouse embryonic fibroblasts (18.78 mg/mL), suggesting a weak potential for embryonic developmental toxicity based on the embryonic stem cell test linear discriminant formula (Wang et al., 2019).

## 6 Critical discussion on the taxonomic status of *Ligusticum chuanxiong*

Based upon more recent botanical literature, WCVP recognises the plant employed as CX as *C. anthriscoides* ‘Chuanxiong’ a variety of the species *C. anthriscoides* (H.Boissieu) Pimenov and Kljuykov. It cites a single scientific synonym for this variety: *Ligusticum chuanxiong* S.H.Qiu, Y.Q.Zeng, K.Y.Pan, Y.C.Tang and J.M.Xu. Medicinal Plant Names Services records 18 non-scientific names in use in the medical literature for drugs derived from this plant drawn from 24 different medical references (https://mpns.science.kew.org/mpns-portal/plantDetail?plantId=2934837&query=conioselinum+anthriscoides&filter=&fuzzy=false&nameType=all&dbs=wcsCmp).

According to WCVP, the species *C. anthriscoides* belongs to the Genus *Conioselinum* which is in the *Apiaceae* family (APG IV classification). WCVP lists 11 scientific synonyms for this species (https://powo.science.kew.org/taxon/urn:lsid:ipni.org:names:77146159-1#synonyms). Globally WCVP lists 18 species belonging to the Genus *Conioselinum* Fisch. ex Hoffm. and a further 37 species belonging to the Genus *Ligusticum L* (in the same family). Regardless of the name used for this species, the 18 species placed by WCVP into the same Genus indicate where the closer evolutionary relationships exist and therefore where there is a greater likelihood of shared chemical pathways. The understanding of taxonomic relationships evolves as more evidence (chemical or molecular) becomes available and will reflect regional access and disciplinary tradition. As a consequence the names employed in different disciplines or at different times will also vary.

Currently, there remain certain discrepancies among researchers regarding the classification of CX whole plant. Yuan et al. (2021) were the first to reveal that the phylogenetic relationship between *Ligusticum chuanxiong* and *Ligusticum jeholense* is closer than that with *Ligusticum sinense*. Consequently, they endorsed the original nomenclature proposed by Qiu for *Ligusticum chuanxiong*, rather than the revision suggested by Pu. Similarly, a 2009 Master’s thesis noted that a comparative study of *Ligusticum chuanxiong* from Sichuan, Gansu, and Yunnan, and its closely related species *Ligusticum sinense* Oliv., revealed morphological, chemical, and chromosomal differences. The study suggested that the *Ligusticum chuanxiong* from Gansu and Yunnan closely resembles the medicinal CX, and the differences in plant morphology and medicinal properties might be attributed to regional, ecological, and anthropogenic factors. In contrast, *Ligusticum sinense* Oliv. showed substantial differences and should be classified as a separate species (Ma, 2009). Furthermore, Ren et al.'s chloroplast phylogenomic analysis emphasized the need to narrow the classification scope of Chinese *Ligusticum chuanxiong*, calling for urgent revisions to its taxonomy (Ren et al., 2022).

In this study, the scientific name used is classified as “unplaced” in authoritative plant taxonomy literature (Govaerts et al., 2021). However, the authors still consider its application in this research to be reasonable. Firstly, the authors focus on the clinical application of CX in China, particularly based on its plant source from the Chinese Pharmacopoeia. The pharmacological effects and clinical applications of CX in CCVDs are summarized and analyzed in detail. CX is a medicinal botanical drug native to Sichuan, China, which is considered a “authentic” medicinal material (authentic medicinal materials refer to those that have been long selected and optimized through clinical application in TCM, grown in specific regions, and distinguished from other materials of the same species by superior quality, efficacy, and stability, and are well-recognized) (Linying et al., 2021). The scientific name *Ligusticum chuanxiong* S.H.Qiu, Y.Q.Zeng, K.Y.Pan, Y.C.Tang and J.M.Xu also appears in the *United States Pharmacopeia*, with a specific reference to its origin from “Sichuan,” confirming it as the plant referred to in this study. Moreover, in March 2024, the International Organization for Standardization (ISO) established a standard for Chinese CX (ISO 8071:2024) (Standardization, 2024), which defines the scope as Ligusticum chuanxiong rhizome (rhizome of *Ligusticum chuanxiong* Hort.), further validating the legitimacy of this scientific name.

However, it is important to note that the taxonomic status of CX whole plant remains an area worthy of further investigation, particularly regarding its relationship with wild populations in terms of morphological, chemical, and genetic characteristics, which has yet to be fully elucidated. Although this study has drawn significant conclusions based on existing literature and experimental data, the complexity of taxonomic classification suggests that the term *Ligusticum chuanxiong* used in different studies may refer to distinct species, varieties, or even different Genus. This potential heterogeneity may influence the comparability and reproducibility of the research results. Additionally, some of the referenced studies did not provide voucher specimen [13 pharmacological studies did provide such data (Yan et al., 2022; Qi et al., 2024; Tang et al., 2021; Yang et al., 2020; ManLi et al., 2023; Feng R. et al., 2023; Juan et al., 2022; Zhong-Hui et al., 2019; Shaojie et al., 2021; Wang et al., 2020; Lim et al., 2024; Pu et al., 2019b; Wu et al., 2021)], highlighting the need for greater emphasis on the standardization and traceability of materials in future research. The conclusions of this study are based on available data, but due to the ongoing taxonomic controversy, future investigations could further validate and refine these findings through more rigorous taxonomic identification and material standardization. Overall, this study provides an important foundation for understanding the pharmacological effects of CX and points the way for future improvements in research.

## 7 Conclusion and prospects

CX, a traditional Chinese medicinal botanical drug, exhibits multiple pharmacological effects, including promoting blood circulation (活血 huoxue), anti-inflammatory, antioxidant, and analgesic activities, and its active components and modern formulations have made it an important agent for the prevention and treatment of cardiovascular diseases and tumors. However, CX also presents the following research limitations: (1) Unclear Taxonomic Status: The distinction between *Ligusticum chuanxiong* and its wild populations (which may be considered different species or subspecies) has not been fully explored. We advocate for multidisciplinary collaboration, involving botanists, chemists, and pharmacologists, to conduct a comprehensive morphological, chemical, and genetic comparative study of *Ligusticum chuanxiong* and its closely related species. This also highlights the need to full describe from where the material was sourced, who authenticated it and where voucher specimens have been consulted. Aubtors should consult https://ga-online.org/best-practice/. Additionally, we recommend that future studies adhere to the standards outlined in the ConPhyMP guidelines to further enhance the scientific rigor and reliability of the research (Heinrich et al., 2022). Only by clarifying its taxonomic status can the scientific validity and reproducibility of future studies be ensured, thereby providing a more reliable theoretical foundation for the clinical application of this medicinal botanical drug. (2) Limitations of Animal Models: The therapeutic effects of CX in disease treatment have primarily been investigated in animal or cell models. These models do not accurately reflect the pathophysiological impact of multiple comorbidities in humans, and translating laboratory findings into clinical practice remains a critical unresolved issue. Consequently, advancing large-scale clinical trials is an essential step to further research and validate the effects of CX. (3) Lack of Bioinformatics Research: Although studies on the metabolites and pharmacological effects of CX have progressively increased, bioinformatics research remains relatively underdeveloped. Bioinformatics, an emerging research methodology, can integrate diverse biological data through computational analysis to elucidate the mechanisms, targets, and relationships between drugs and diseases. When confronted with the limitations of clinical or animal research, network pharmacology studies of CX may provide valuable insights. By constructing the interaction network between drugs and diseases, network pharmacology helps identify potential targets of CX metabolites, explore their pathways in complex biological systems, and further enrich and broaden the scope of traditional pharmacological research. (4) Unresolved Mechanisms of Action: Although several studies have explored the mechanisms of action of CX, a unified consensus has yet to emerge within the academic community. Elucidating the mechanisms of CX will enable a more accurate assessment of its safety and efficacy. (5) Delayed Product Development: Currently, CX products remain limited. The CX-related drugs marketed in China primarily consist of CX tea powder, with limited products developed through the compatibility of CX and its active metabolites with other drugs, such as Danggui and Huangqi *[Astragalus membranaceus (Fisch.) Bge. var.mongholicus (Bge.) Hsiao]*. The national government has yet to prioritize the development of relevant innovative products. (6) Neuropharmacological Potential Yet to Be Explored: Although some literature suggests limited pharmacological properties of CX in the nervous system, the research is still in its preliminary stages. Nevertheless, CX holds significant research potential in neuroprotection and antidepressant effects. In conclusion, CX is a multifunctional compound with vast research potential. Future research will facilitate the advancement of its development in broader applications, particularly in the treatment of neurological diseases.

## Glossary

Aβ: amyloid-β protein
AD: Alzheimer’s Disease
AKI: acute kidney injury
ALI: acute lung injury
ALS: Amyotrophic lateral sclerosis
ARDS: acute respiratory distress syndrome
AS: atherosclerosis
ATP: adenosine triphosphate
AMPK: adenosine 5‘-monophosphate (AMP)-activated protein kinase
AQP: aquaporin 4
AK2: Janus kinase 2
Bcl-2: B-cell lymphoma-2
BKCa: big potassium calcium-activated channels
Bax: Bcl-2 associated X protein
CX: dried rhizome of *Ligusticum chuanxiong*
CCVD: cardio-cerebral vascular diseases
CUL4B: Cullin 4B
CCI: chronic compression injury
c-JUN: c-Jun N-terminal kinase
Cx43: connexin43
CXCL12: chemokine C-X-C motif ligand 12
CXCR4: chemokine C-X-C motif receptor 4
Caspase-1: cysteine-requiring aspartate protease-1
caspase-3: cysteinyl aspartate specific proteinase 3
COX2: cyclooxygenase-2
caspase-8: cysteine-requiring aspartate protease-8
CCR1: C-C Motif Chemokine Receptor 1
DN: diabetic nephropathy
DNA: deoxyriboNucleic Acid
EO: essential oil
ERK: extracellular signal-regulated kinase
FAK: focal adhesion kinase
FGF: fibroblast growth factor
FGFR3: fibroblast growth factor receptor 3
GSK: Glycogen Synthase Kinase
GTP: guanosine triphosphate
GPX4: glutathione peroxidase 4
GSDMD: gasdermin D
HD: Huntington’s disease
HO-1: heme oxygenase-1
HSC: hepatic stellate cell
H3K9ac: histone 3 lysine 9 acetylation
hNOS: human neuronal oxidative stress
IL: Interleukin
I/R: ischemia/reperfusion
iNOS: inducible nitric oxide synthase
ISO: International Organization for Standardization
KAT2A: lysine acetyltransferase 2A
LCP: ligusticum CX polysaccharides
LKB1: liver kinase B1
LIMK1: LIM Domain Kinase 1
LD50: median lethal dose
MAPK: mitogen-activated protein kinase
MCPIP1: monocyte chemotactic protein-induced protein 1
ME: microsphere-embolized
MMP: matrix metalloprotein
mTOR: mammalian target of rapamycin
NFTs: neurofibrillary tangles
NMPA: National Medical Products Administration
NO: nitric oxide
Nrf2: nuclear factor erythroid 2-related factor 2
NFκB: nuclear factor kappa-B
NADPH: nicotinamide adenine dinucleotide phosphate
NOD2: nucleotide-binding oligomerization domain 2
OGD/R: oxygen-glucose deprivation/reperfusion
NLRP3: Nod-like receptor family pyrin domain-containing 3
PD: Parkinson’s disease
PKC: protein kinase C
PI3K: phosphoinositide 3-kinase
PPAR: peroxisome proliferator-activated receptors
PINK1: PTEN induced putative kinase 1
Prx1: paired related homeobox 1
PGC-1α: peroxisome proliferator-activated receptor-γ coactivator-1α
PKC-β: protein kinase C beta
PAI-1: plasminogen activator inhibitor-1
QBT: a novel phthalein metabolite
Rac1: Rac Family Small GTPase 1
SMCs: smooth muscle cells
SR: scavenger receptor
SREBPs: sterol regulatory element-binding proteins
STZ: streptozotocin
SOD: superoxide dismutase
siRNA: small interfering RNA
STAT3: signal transducer and activator of transcription 3
SSRT4: somatostatin receptor 4
SIRT-1: silent information regulator 1
SCAP: sterol regulatory element-binding proteins leavage activating protein
TBN: tetramethylpyrazine nitrone
TCM: traditional Chinese medicine
TMP: tetramethylpyrazine
TNF: tumor necrosis factor
TRAF6: tumor necrosis factor receptor-associated factor 6
TLR4: Toll-like receptor 4
TDP-43: TAR DNA-binding protein of 43 kDa;
WCVP: World Checklist of Vascular Plants
xCT: cystine-glutamate antiporter
ZO-1: Zonula occludens-1
3-NP: 3-Nitropropionic acid
5-HIAA: 5-hydroxyindole acetic acid
5-HT: 5-hydroxytryptamine
5-HT1B: 5-Hydroxytryptamine Receptor 1B
